# Ultrastructural Alterations of the Glomerular Filtration Barrier in Fish Experimentally Exposed to Perfluorooctanoic Acid

**DOI:** 10.3390/ijerph20075253

**Published:** 2023-03-24

**Authors:** Maurizio Manera, Fabio Casciano, Luisa Giari

**Affiliations:** 1 Department of Biosciences, Food and Environmental Technologies, University of Teramo, St. R. Balzarini 1, 64100 Teramo, Italy; 2 Department of Translational Medicine and LTTA Centre, University of Ferrara, St. Fossato di Mortara 70, 44121 Ferrara, Italy; 3 Department of Environmental and Prevention Sciences, University of Ferrara, St. Borsari 46, 44121 Ferrara, Italy

**Keywords:** kidney, toxicologic pathology, environmental pathology, fish model, glomerular protein leakage, per- and polyfluoroalkyl substances

## Abstract

Per- and polyfluoroalkyl substances can be referred to as the most critical group of contaminants of emerging concern. They can accumulate in high concentration in the kidney and are known to potentially affect its function. Nonetheless, there is a lack of knowledge about their morphopathological effect on the glomerular filtration barrier. Since previous research suggests perfluorooctanoic acid (PFOA) induces glomerular protein leakage, the glomerular filtration barrier of 30 carp from the same parental stock (10 unexposed; 10 exposed to 200 ng L^−1^ of PFOA; and 10 exposed to 2 mg L^−1^ of PFOA for 56 days) was screened for possible PFOA-induced ultrastructural lesions in order to shed light on the related pathophysiology. PFOA exposure affected the glomerular filtration barrier in carp experimentally exposed to 2 mg L^−1^, showing ultrastructural alterations compatible with glomerulonephrosis: podocyte effacement, reduction of filtration slits and filtration slit diaphragms, basement membrane disarrangement, and occurrence of proteinaceous material in the urinary space. The results of the present research confirm the glomerular origin of the PFOA-induced protein leakage and can contribute to the mechanistic comprehension of PFOA’s impact on renal function and to the assessment of the exposure effect of environmental pollutants on animals and humans, according to the One Health approach.

## 1. Introduction

Despite the structural diversity of excretory organs among metazoan taxa, two morphofunctional excretory compartments can be identified: the primary urine-producing apparatus and the modulating tubule [1]. Notably, podocyte-based metanephridial systems are the primary urine-producing apparatus of eucoelomates, including vertebrates, where podocyte basic architecture is phylogenetically conserved [1]. Exclusive to vertebrates are the more developed primary processes in the podocyte, the presence of glomerular capillary loops, lined by fenestrated endothelial cells and the occurrence of mesangial cells, specialized pericytes [1,2]. Accordingly, the glomerular filtration barrier is mainly constituted by the vascular (fenestrated endothelial cells and mesangial cells) and epithelial (podocytes) compartments and the interposed basement membrane [1]. Such basic architecture is completed by the presence of glycocalyx on fenestrated endothelial cells, but also between podocytes and basement membrane, and of a slit diaphragm between contiguous pedicels, mainly formed by the transmembrane protein nephrin [3,4,5,6,7]. Since the morphofunctional integrity of the glomerular filtration barrier is of paramount importance to grant proper renal excretory functionality and all the integrated/related functions, it has been extensively studied for possible morphofunctional alteration, with particular regard to proteinuria (urine protein leakage) [4,8,9,10,11].

Among other water pollutants, contaminants of emerging concern (CECs) are particularly relevant because they may cause ecological, wildlife, and human health impacts because they are not fully regulated and/or not routinely monitored in the environment [12]. Per- and polyfluoroalkyl substances (PFAS) can be referred to as the most critical group of CECs [13]; they can accumulate at high concentration in the kidney, playing a primary role in their excretion, and are known to potentially affect renal function [14,15,16,17,18,19,20,21,22,23,24]. Nonetheless, there is a generalized lack of knowledge about the morphopathological and ultrastructural effects of these pollutants on the glomerular filtration barrier [14,15,16,17,18,19,20,21,22,23,24]. PFAS represent a heterogeneous class of synthetic fluorinated chemicals, containing at least one fully fluorinated methyl or methylene carbon atom [25]. PFAS have been widely used in many industrial processes and products (e.g., fire-suppressing foams, food packaging, textile treatments) [26,27,28]. They are released into the environment during industrial production, commercial use, final usage, and disposal, and being extremely stable and mobile in both abiotic and biotic matrices, they are considered ubiquitous contaminants, belonging to the group of persistent organic pollutants (POPs) [29,30]. Perfluorooctanoic acid (PFOA), one of the best-known PFAS, shows amphiphilicity, having a hydrophobic 7-carbon chain in which hydrogen atoms are substituted by fluorine atoms and a hydrophilic carboxylic group [31]. PFAS are of high concern for aquatic ecosystems due to their strong water solubility, persistence, and long half-life in organisms [32,33]. Recently, the European Commission amended Annex I to Regulation (EU) No. 2019/1021 on persistent organic pollutants (POPs Regulation) to ban PFOA, its salts, and PFOA-related compounds [34]. More recently, the U.S. Environmental Protection Agency has issued interim updated drinking water health advisories for PFOA and perfluorooctane sulfonic acid (PFOS) [35]. In particular, the interim updated health advisories for PFOA are 0.004 ppt, which is 10^−3^ of the detection limit of the currently approved analytical method for PFOA, posing significant challenges in developing ultrasensitive analytical methods [36,37]. These stresses underscore the worldwide concern about the effects of PFAS in general and PFOA in particular.

Fish are certainly the best candidates for aquatic ecosystem monitoring purposes, completing all their life cycle in water and being the most representative vertebrates [38]. With regard to PFAS, most fish studies have been performed on cyprinids, especially *Danio rerio* (Hamilton, 1822) [39]. In spite of the unquestionable success and usefulness of *D. rerio* in biomedical research, common carp (*Cyprinus carpio* Linnaeus, 1758) should be regarded as an animal model both for field and experimental studies on PFAS due to its widespread and abundant presence in freshwater ecosystems worldwide, the fact that it is easy to rear and maintain in captivity, the fact that it is a food source, and the fact that it has been considered an “ecological engineer” [22,40,41,42]. It is worth mentioning that animals may be used according to the One Health approach, both as experimental models in biomedical research and as indicator species for assessing the exposure effect of environmental pollutants on humans and animals [43,44]. Common carp has already been indicated as a sentinel fish for PFOA-induced damages and as a related fish model [45].

The effect of PFOA on the nephron modulator tubular compartment has previously been tested in the same fish as the present study [22]. Ultrastructural evidence of glomerular protein leakage was deduced from the increased glomerular protein ultrafiltrate pinocytosis by epithelial cells in the first proximal tubular segment. Apart from glomerular capillary bed dilation and reduction of the normal glomerular folding pattern, no appreciable pathological changes were noted at light microscopy, despite the observation of epoxy resin-embedded semithin sections [22]. Though these alterations were particularly evident in specimens from fish exposed to the highest tested concentration (2 mg L^−1^), they were also present, as incipient features, in fish exposed to the lowest tested concentration (200 ng L^−1^), despite the fact that the analytical PFOA renal concentration was below the limit of detection (LOD) [22]. The apparent discrepancy between morphopathological but also gene expression results and the related analytical PFOA tissue concentration under the LOD in carp exposed to 200 ng L^−1^ PFOA has also been observed and discussed for the liver, the renal hemopoietic tissue, the gonads, and the renal thyroid follicles from the same carp as the present research, stressing the possible limitation/bias in the adoption of PFOA analytical detection in tissues as a biomarker of exposure [22,45,46,47].

Though light microscopy should be regarded as the first line of investigation in pathology, the adoption of transmission electron microscopy should be considered mandatory in renal pathology, with particular regard to the study of the glomerular filtration barrier [11,48,49,50]. Therefore, the carp from the previous research [22], experimentally exposed to two PFOA concentrations (200 ng L^−1^ and 2 mg L^−1^) for 56 days, were specifically screened for ultrastructural lesions at the glomerular filtration barrier level in comparison to the unexposed ones, in order to shed light on the possible PFOA-induced glomerular protein leakage pathophysiology.

Perfluorooctanoic acid exposure was shown to affect the glomerular filtration barrier in all three of its main components (fenestrated endothelium, basement membrane, and podocyte) in carp experimentally exposed to 2 mg L^−1^, showing ultrastructural signs compatible with the diagnosis of glomerulonephrosis. Accordingly, this is the first experimental study documenting PFOA-induced lesions at the glomerular filtration barrier and can contribute to the mechanistic comprehension of PFOA’s impact on renal function, with particular regard to glomerular protein leakage, and the assessment of the exposure effect of environmental pollutants to animals and humans, according to the One Health approach.

## 2. Materials and Methods

The renal samples examined during the present study were obtained from a previous one [51], where 31 two-year-old common carp (total length: 19.32 ± 2.49 cm, body mass: 104.84 ± 27.80 g [mean ± standard deviation]) from the same parental stock were experimentally exposed to two PFOA (PFOA standard, chemical purity 96%, Sigma-Aldrich catalogue number 171468, Merck KGaA, Darmstadt, Germany) concentrations (200 ng L^−1^ [n = 10] and 2 mg L^−1^ [n = 11]) for 56 days, coherently with a sub-chronic exposure, in a flow-through open system (500 mL of water min^−1^) and tissues examined for possible induced alteration in comparison to unexposed, control fish (n = 10). In particular, the dose of 200 ng L^−1^ was adopted as an environmentally relevant concentration based on PFOA reports in surface water [52], whereas the dose of 2 mg L^−1^ was chosen to induce a certain histological response, as previously reported in other cyprinid fish [53]. Fish were euthanized by spinal cord severing after an anesthesia overdose with tricaine methanesulfonate (MS-222). Tissue PFOA concentrations were found to be below the limit of detection (LOD = 0.4 ng g^−1^), using high performance liquid chromatography with electrospray ionization tandem mass spectrometry, in fish exposed to the lowest concentration (200 ng L^−1^), while PFOA concentrations in fish from the highest tested concentration (2 mg L^−1^) were 64.87 ± 24.25 in blood and 1.08 ± 0.54 in kidney (ng g^−1^ wet weight, mean ± standard deviation) [51]. The reader is referred to Giari et al. (2016) [51] for further details about the experimental design, fish biometry, and analytical quantification of PFOA concentrations in tissues/organs, and to Manera et al. (2021, 2022) [22,45,54] for electron microscopy technique.

In brief, referring only to the topic of the present study, 30 representative kidney samples from 30 carp (10 unexposed, 10 exposed to 200 ng L^−1^ of PFOA, and 10 exposed to 2 mg L^−1^ of PFOA) were collected and processed for electron microscopy as follows: Samples were fixed in 2.5% glutaraldehyde buffered with sodium cacodylate (pH 7.3) at 4 °C for 3 h, post-fixed in 1% osmium tetroxide for 2 h, dehydrated in a graded series of acetone, and embedded in epoxy resin (Durcupan™ ACM, Fluka, Sigma-Aldrich, St. Louis, MO, USA). Ultrathin sections (90 nm) were contrasted with uranyl acetate and lead citrate and examined under a Zeiss EM 910 transmission electron microscope (Carl Zeiss Microscopy GmbH, Oberkochen, Germany) operating at 120 kV.

## 3. Results

**Figure 1:**
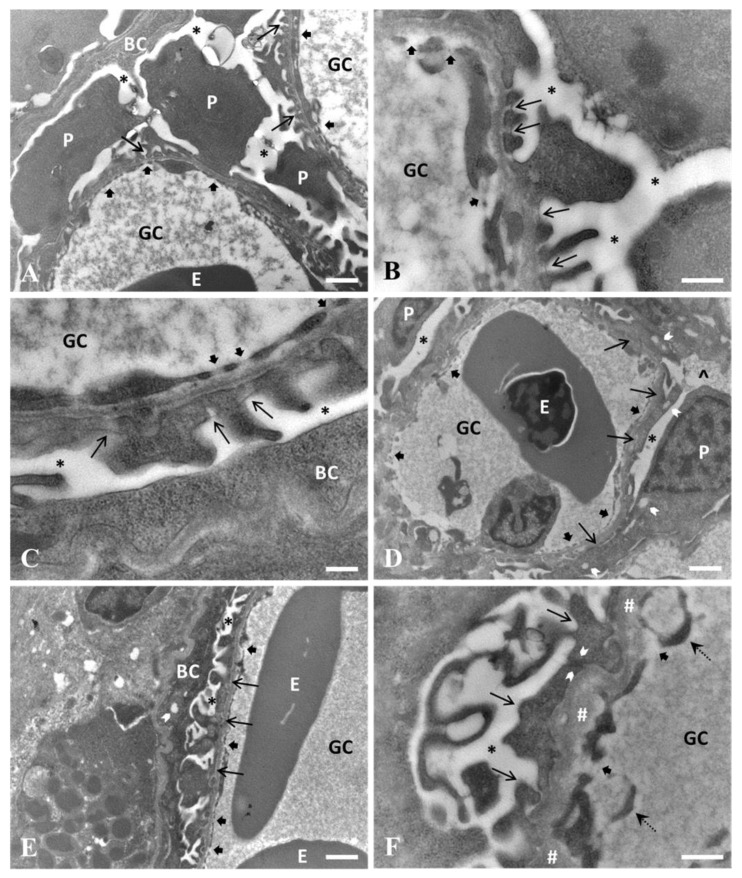
Transmission electron micrographs of the glomerular filtration barrier in unexposed carp (**A**,**B**), carp exposed to PFOA 200 ng L^−1^ (**C**), and carp exposed to 2 mg L^−1^ (**D**–**F**). (**A**) The normal architecture of the glomerular filtration barrier is shown. The large bodies of podocytes (P) are appreciable, along with the typical interdigitated finger-like tertiary foot processes (pedicels) (thin arrows), contacting the underlying basement membrane, interposed between the foot processes and the fenestrated endothelium (large arrows) of glomerular capillaries (GC). (BC) Bowman’s capsular epithelium. (E) Erythrocyte. (*) Urinary space. Scale bar = 1 µm. (**B**) The slit diaphragm is appreciable as a fine electron-dense line bridging contiguous pedicels (thin arrows) in correspondence of each filtration slit. The typical fenestrae (large arrows) of the fenestrated endothelium of glomerular capillaries (GC) are visible. (*) Urinary space. Scale bar = 0.5 µm. (**C**) The glomerular filtration barrier of a carp exposed to the lowest tested concentration (200 ng L^−1^) shows a substantially maintained architecture (cf. (**A**)), with particular regard to the relative number of filtration slits and the integrity of slit diaphragms (thin arrows) and fenestrae (large arrows) of the fenestrated endothelium of glomerular capillaries (GC). (BC) Bowman’s capsular epithelium. (*) Urinary space. Scale bar = 0.25 µm. (**D**) The glomerular filtration barrier shows podocyte effacement, with a drastic reduction in the number of filtration slits, the fusion of pedicels, and the occurrence of a large, continuous cytoplasmic sheet (thin arrows). Focal vacuolations are visible in the podocyte (P) cell body and processes (arrowheads). Furthermore, foamy proteinaceous material (^) is observable in the urinary space (*). (E) Erythrocyte. (GC) glomerular capillary. (Large arrows) fenestrated endothelium. Scale bar = 1 µm. (**E**) Evidence of foot process fusion (thin arrows) is appreciable with the disappearance of filtration slits in the glomerular filtration barrier. A single focal vacuolation (arrowhead) is observable in an epithelial cell of Bowman’s capsule (BC). (*) Urinary space. (E) Erythrocyte. (GC) glomerular capillary. (Large arrows) fenestrated endothelium. Scale bar = 1 µm. (**F**) At higher magnification, pedicles (thin arrows) appear distorted, enlarged, and filtration slits drastically reduced, compared to unexposed carp (cf. (**B**)) and carp exposed to the lowest tested concentration (cf. (**C**)). There is no evidence of slit diaphragms in the occasionally present filtration slits. Evidence of close contact is appreciable between the cell membranes of two contiguous pedicels (arrowheads). Disarrangement and enlargement of the basement membrane (#) are also present compared to the carp of the other two experimental groups (cf. (**B**,**C**)). Furthermore, irregular villous-like cytoplasmic projections are visible (dotted arrows). (Large arrows) fenestrae of fenestrated endothelium. (*) Urinary space. (GC) glomerular capillary. Scale bar = 0.4 µm.

Unexposed fish showed the typical renal corpuscle structure found in vertebrates, where podocytes, fenestrated endothelial cells, and the interposed basement membrane constitute the glomerular filtration barrier. Podocytes showed a large cell body with elongated branching cell processes (primary and secondary processes) ending in fine interdigitated finger-like tertiary foot processes, also known as pedicels (average width = 228 nm), contacting the basement membrane (average thickness = 146 nm) (Figure 1A). Contiguous pedicels displayed interposed gaps where the basement membrane was not covered by the aforementioned podocyte processes, thus forming the filtration slits (average width = 82 nm). In correspondence with each filtration slit, a filtration slit diaphragm was appreciable as a fine, electron-dense line bridging contiguous pedicels (Figure 1B). Glomerular endothelial cells appeared as elongated, flattened cells, displaying the characteristic transcellular perforations known as fenestrae (average width = 136 nm) (Figure 1A,B).

The glomerular filtration barrier was affected by PFOA exposure, but only at the highest tested concentration (2 mg L^−1^), whereas the ultrastructural architecture was maintained in fish exposed to the lowest tested dose (200 ng L^−1^) (Figure 1C). At the highest dose, podocyte effacement occurred with retraction, widening, disarrangement, and fusion of the normally interlocked finger-like pedicles, which were substituted by a continuous cytoplasmic sheet (average width = 1713 nm) and/or pleomorphic, deformed pedicles (average width = 286 nm) (Figure 1D). As a consequence, there was a drastic reduction in the number of filtration slits and filtration slit diaphragms and a general disarrangement and relative enlargement of the basement membrane (average thickness = 237 nm) (Figure 1E,F). Underlying fenestrated endothelial cells showed slightly enlarged fenestrae (average width = 180 nm) and irregular villous-like cytoplasmic projections (Figure 1F). Moreover, focal cytoplasmic vacuolations were observed in podocyte processes, and foamy, proteinaceous material was detected in the urinary space (Figure 1D).

## 4. Discussion

The architecture of the glomerular filtration barrier of unexposed carp agrees with previous studies in eucoelomates and vertebrates, in general, and in commom carp, in particular [1,55,56].

Perfluorooctanoic acid exposure was shown to affect the glomerular filtration barrier in all three of its main components (fenestrated endothelium, basement membrane, and podocyte) in carp experimentally exposed to 2 mg L^−1^, promoting the outflow of proteinaceous material in the urinary space, hence confirming the glomerular origin of the protein leakage reported in a previous study on the same fish [22]. The pathophysiology of proteinuria (urine protein leakage) continues to intrigue researchers, focusing on the glomerular filtration barrier and/or proximal tubule integrity. With regard to the glomerular filtration barrier, all the constitutive components may be altered to a various degree and through possible reciprocal association due to the cross-talk between the main cellular components: fenestrated endothelial cells and podocytes [5,7,57,58,59,60]. As the first component of the glomerular filtration barrier, the glycocalyx is known to function as a negatively charged ion sieve, making it particularly efficient to prevent/reduce albumin leakage through the glomerular filtration barrier. As a consequence, its alteration is considered to be an incipient cause of proteinuria [4,5,57,61]. Unfortunately, glycocalyx requires dedicated processing/staining techniques to ensure its best evaluation under transmission electron microscopy [62,63], and because specimens were processed routinely for the present study, it was not possible to rule out possible alterations of glycocalyx related to PFOA exposure and its possible contribution to proteinuria. More recently, transcytosis by both the fenestrated endothelial cells and the podocytes has been proposed to be involved in the pathogenesis of albuminuria (urine albumin leakage), also without glycocalyx alteration, overtaking the conventional theory of “impairment of the size- and/or charge-selective filtration barrier” [60,64,65]. As a consequence, the possible role of glycocalyx and transcytosis should be addressed in further studies, with particular regard to the lowest, environmentally relevant PFOA concentration (200 ng L^−1^), where no ultrastructural evidence of alteration of the glomerular filtration barrier was noted. Referring to the highest tested PFOA concentration (2 mg L^−1^), the reported architecture disarrangement agrees with the known morphopathological evidences of glomerulonephrosis (i.e., the morphofunctional alteration of the glomerular filtration barrier), with particular regard to podocyte effacement [9,11,48,58,66,67]. Though PFOA and other PFAS can accumulate at high concentrations in the kidney and potentially affect renal function, no previous research has specifically addressed the effect of these pollutants on the glomerular filtration barrier [14,15,16,17,18,19,20,21,22,23,24], making it difficult to interpret and compare the observed alterations and speculate about the possible underlying pathophysiology. Interestingly, none of the lesions seen in other anatomical districts (namely mitochondrial cristolysis, vesiculation, swelling, and ballooning, autophagosomes occurrence, rough endoplasmic reticulum degranulation, disarrangement and enlargement in hepatocytes [54]; increased number and volume of cytoplasm vesiculations in cells of the first proximal tubular segment, mitochondrial focal vesiculation in cells of the distal tubular segment and of the collecting ducts in kidney [22]; rough endoplasmic reticulum enlargement and fragmentation, cytoplasm vacuolation, enhanced phagolysosomes formation in thyroid follicles [45]) were appreciated at the level of glomerular filtration barrier, suggesting a somewhat different pathogenesis compared to the previous tissues. Given the ultrastructural alterations documented during the present study, the plasma membrane, cytoskeleton, and adhesion molecules should be considered as possible targets of PFOA-induced damages. Indeed, PFOA and other PFAS have been shown to alter plasma membrane potential and to acidify cytosol in a human colon carcinoma HCT116 cell model due to their amphipatic structure, suggesting these alterations may precede reactive oxygen species production and mitochondrial transmembrane potential impairment [68]. Moreover, membrane potential dysregulation and alteration of the organization of membrane microdomains have been reported in boar spermatozoa experimentally exposed to PFOA and perfluorooctane sulfonate (PFOS) [69]. Recently, exposure of human HepaRG hepatoma cells to PFOA and PFOS has resulted in altered bile canalicular structure and bile flow impairment, caused by actin cytoskeleton disarrangement and to structural redistribution of the tight-junctional protein ZO-1 [70]. More information on the effects of PFOA and PFOS on F-actin, actin binding proteins, and adhesion molecules is available in Wang et al. [71]. Actin filament remodeling and an increase in endothelial permeability have been reported in human microvascular endothelial cells exposed to PFOS as a consequence of reactive oxygen species production [72]. The integrity of the plasma membrane, cytoskeleton, and adhesion molecules is of paramount importance in the maintenance of a proper and functional structure of the glomerular filtration barrier [5,10,11,61,73,74]. As a consequence, further studies should specifically address the topic with particular regard to the effect of PFOA and other PFAS on the fenestrated endothelium and on podocytes, both affected during the present study.

In the carp of the present study, the highest PFOA concentration was found in blood [51], the same as in other studies on fish and other vertebrates, though differences may arise according to the route of contamination, dosage/concentration, duration of exposure, and other biological parameters [18,20,23]. Worth noting is that PFAS bind to albumin and other blood proteins [23,75,76], so following the albumin route across the glomerular filtration barrier may contribute to shedding light on the underlying pathogenesis. Irrespective of how albumin can transit across the endothelial (e.g., transcytosis) and basement membrane barriers, it can be internalized by podocytes, partially bypassing the filtration slit diaphragm through transcytosis [77], allowing PFOA to enter the cells that are critical for maintenance of the morphofunctional integrity of the glomerular filtration barrier. Moreover, in humans, PFOA is known to be filtered freely in the glomerulus, actively excreted in the proximal tubule, and then readsorbed by organic anion transport (OAT) peptides [78,79]. Interestingly, in the mouse, kidney OAT-like peptides have been described in blood vessels, parietal epithelial cells, podocytes, distal convoluted tubules, connecting tubules, and collecting tubules [80]. The role of small molecule membrane transporters found in the mammalian podocyte in glomerular pathogenesis and as a possible therapeutic target has been discussed by Zennaro et al. (2014) [81]. OAT peptides are phylogenetically conserved and present in zebrafish, where marked variation according to tissue and sex has been reported [82,83]. As previously stressed by Manera et al. (2022), and differently from humans, where nephron segments are known to be differentially affected by toxicants, according to the implied transporter and its possible sex- and genetic-based modifications, there is a generalized lack of knowledge about nephron topographic toxicologic pathology for fish [22].

As a possible alternative/complementary pathogenesis, the effect on the glomerular filtration barrier of PFOA-induced hypertension should be considered, at the light of the histological signs of glomerular hyperperfusion observed in a previous study on the same carp [22]. In such a case, podocyte damage would be secondary to glomerular capillary dilation, as proposed by Kriz et al. (2022) in two hypertensive rat models of focal and segmental glomerulosclerosis [84]. Nevertheless, the association of PFAS with hypertension is still a matter of debate, with some studies claiming it and others rejecting it [85,86,87].

It is worth mentioning that pathology, as a discipline, relies on lesions to formulate a diagnosis, a lesion being the morphological evidence (at any integration level) of a disrupted function [88], providing precious information to elucidate the underlying pathogenesis. Furthermore, current nephropathological diagnostic guidelines, with particular regard to the glomerular filtration barrier, rely on qualitative detection and evaluation of codified ultrastructural alterations [11,48,49,50]. Nevertheless, and in spite of the paramount importance of electron microscopy to assess toxicity [89], further targeted studies are needed to elucidate at best the likely pathogenesis of PFOA at the glomerular filtration membrane level.

## 5. Conclusions

Perfluorooctanoic acid exposure was shown to affect the glomerular filtration barrier in carp experimentally exposed for 56 days at 2 mg L^−1^. Fenestrated endothelium, basement membrane, and podocytes showed ultrastructural lesions compatible with glomerulonephrosis, confirming the glomerular origin of the PFOA-induced protein leakage. The underlying pathogenesis needs to be elucidated with further targeted studies addressing PFOA’s effect on the plasma membrane, cytoskeleton, and adhesion molecules at the glomerular filtration barrier level, along with toxicological studies specifically addressing the possible implications in terms of toxicodynamics and toxicokinetics. The results of the present research can contribute to the mechanistic comprehension of PFOA’s impact on renal function, with particular regard to glomerular protein leakage, and can be used to assess the exposure effect of environmental pollutants on animals and humans, according to the One Health approach.

## Data Availability

Since the study relies on qualitative detection and evaluation of codified ultrastructural alterations, the relevant qualitative data (figures) are reported within the present article.
